# Quality of Life Assessment After Uterine Artery Embolization in Patients with Fibroids Treated in an Ambulatory Setting

**DOI:** 10.3390/diagnostics15060739

**Published:** 2025-03-16

**Authors:** Jean-Francois Geschwind, Bahman Afsari, Nariman Nezami, Jacob White, Michael Shor, Yan Katsnelson

**Affiliations:** 1 USA Fibroid Centers and Clinics Group, Northbrook, IL 60062, USA; jwhite@usaclinics.com (J.W.); mshor@usaclinics.com (M.S.); ykatsnelson@usaclinics.com (Y.K.); 2 National Cancer Institute, National Institutes of Health, Bethesda, MD 20892, USA; 3 Department of Oncology, Johns Hopkins University, Baltimore, MD 21205, USA; 4 Division of Vascular and Interventional Radiology, Department of Radiology, MedStar Georgetown University Hospital, Washington, DC 20007, USA; nariman.nezami@medstar.net; 5 School of Medicine, Georgetown University Washington, DC 20007, USA; 6 Lombardi Comprehensive Cancer Center, Washington, DC 20007, USA

**Keywords:** uterine fibroid, leiomyomata uteri, uterine artery embolization, quality of life questionnaire, fibroid symptoms, outpatient care, office-based lab

## Abstract

**Background:** Despite the growing acceptance of uterine artery embolization (UAE) to treat women with fibroid disease, its wider use remains limited because it is not considered to be a definitive therapy, as opposed to surgical treatments such as myomectomy or hysterectomy. Given the evolution of health care towards outpatient medicine, it is critical to determine the impact of UAE on the quality of life (QoL) of women with fibroid disease treated in an outpatient setting. **Objectives:** The purpose of this study was to assess the QoL of patients with fibroids treated with UAE in an office-based lab setting. **Study Design:** This prospective single-arm study was approved by the western IRB (wIRB) and included 1285 consecutive patients—the largest study on UAE to date—enrolled from September 2021 to December 2023 who were seen for a baseline evaluation in a clinic and then, subsequently, between 2 and 8 months post-UAE for follow-up clinical and imaging evaluation. Patient QoL was assessed using the validated QoL questionnaire: the Uterine Fibroid Symptom and Health-Related Quality of Life questionnaire. **Results:** The results from all 1285 patients were analyzed. The median and mean follow-up periods were 182 and 180 days, respectively (interquartile range of 19 days). UAE led to reduced bleeding in 96% of patients, pelvic pain and bulk-related symptoms in 94%, fatigue in 94%, and urination frequency in 92%. On the other hand, improvements were seen in the level of activity in 82%, energy and mood in 85%, and sexual function in 71% of the patients, whereas the general QoL index significantly increased in 86% of the patients (*p* < 0.001). More than one third of our patients (39%) had Medicaid insurance, reflecting the relatively low socioeconomic status of our patient population. **Conclusions:** In this largest clinical trial on UAE to date, we found that performing UAE in an outpatient setting significantly improved patients’ clinical symptoms such as bleeding and bulk symptoms and, most importantly, their overall QoL.

## 1. Introduction

Uterine fibroids (leiomyomata uteri) are the most common benign tumor of the uterus, occurring in approximately 20–50% of women of reproductive age [1,2]. Race, age, family history, time since last birth, premenopausal state, hypertension, and diet comprise the known risk factors for fibroid disease [1,2,3,4]. The incidence is significantly higher for Black (80%) compared to Caucasian (70%) women, with nearly a quarter of Black women between the ages of 18 and 30 affected by fibroids (vs. 6% of Caucasian women), climbing to 60% by age 35 [3,4]. Although fibroids are benign tumors, they are responsible for symptoms in approximately half of women with fibroids, including pelvic pain, bulk symptoms of pressure, bleeding both during (menorrhagia) and between (metrorrhagia) menstrual cycles that can cause anemia, day- and nighttime urinary frequency, fatigue, dyspareunia, and infertility [5,6,7,8,9,10]. Although such symptoms may be quite debilitating, prompting women to seek care for their fibroids, many women remain unaware of the health impact of their fibroid-specific symptoms, frequently leading to a long wait before definitive therapy is contemplated [5,6,7,8,9,10]. Treatment standards are typically surgical in nature and take the form of myomectomy, which spares the uterus but has been somewhat plagued by high recurrence rates, or hysterectomy, which some have criticized for being overused [5,6,7]. The emergence of image-guided therapies within the field of interventional radiology has led to the growing acceptance of uterine artery embolization (UAE) as an established therapy for fibroid disease because it leaves the uterus intact while causing involution or, in some instances, complete disappearance of the fibroids [11,12,13,14]. During the nearly 30 years of its existence and its availability to women, many clinical studies and published manuscripts have demonstrated UAE’s safety and efficacy, especially for symptomatic relief [11,12,13,14]. Yet, despite its growing acceptance, resistance to its wider use still exists because UAE is not considered to be a definitive therapy—as opposed to surgical treatments—and because concern about fibroid recurrence remains. Such arguments are largely responsible for the development of fibroid disease-specific quality of life (QoL) measures to determine the clinical success of UAE, especially given the negative impact of fibroid-related symptoms on physical and social activities, general quality of life, and work productivity [15,16]. To that end, the Uterine Fibroid Symptom and Health-Related Quality of Life (UFS-QoL) questionnaire was developed and validated to address such issues [15,16]. Several studies seemed to indicate the positive impact of UAE on patient quality of life (QoL), thereby favoring its use over other more invasive therapies [12,16,17,18]. As medicine is evolving towards outpatient care, which was accelerated by the Covid crisis, access to care has been facilitated and increased for patients, especially those in low socioeconomic urban areas, by the presence of dedicated outpatient facilities, such as ambulatory surgical centers (ASCs) and office-based labs (OBLs) [19]. Physicians working in OBLs are performing increasingly complex procedures for patients with peripheral artery and cardiac disease, as well as other conditions, such as fibroid disease, benign prostatic hypertrophy, and various cancers [19]. Thus, it is critical to study the effect of UAE on the QoL of patients suffering from fibroid disease treated in an outpatient setting, especially given the paucity of data that exists. Therefore, the purpose of our study was to assess the impact of UAE in women with fibroid disease treated in the largest OBL system in the United States on their QoL using the UFS-QoL questionnaire.

## 2. Materials and Methods

### 2.1. Study Design and Patient Population

This prospective study was approved by the wIRB and included 1285 consecutive patients—the largest study conducted on UAE to date—enrolled from September 2021 to December 2023 who were seen for a baseline evaluation in clinic and then, subsequently, between 2 and 8 months post-uterine artery embolization (UAE) for a clinical follow-up and, for some, an imaging evaluation. The median and mean follow-up periods were 182 and 180 days, respectively (interquartile range of 19 days; 25th percentile of 177 days and 75th percentile of 196 days). All patients signed an informed consent form to be included in the study, and no specific exclusion criteria were used, as the study was designed to enroll consecutive patients. Out of 1285 patients, we had the information of 1283 patients, of whom 292 (38.95%), 45 (3.56%), and 726 (57.48%) had Medicaid, Medicare, and Private Health insurance, respectively.

### 2.2. Assessment of Quality of Life

Analysis was performed using a dedicated, fibroid-specific, and validated quality of life (UFS-QoL) questionnaire that has been used in other studies [15,16,20,21,22], consisting of 37 questions about six QoL domains and clinical symptoms. Symptoms and measures of quality of life were compared from baseline to follow-up time points. In total, 8 questions measure clinical symptoms and 29 address health-related quality of life issues. The symptom questions consist of 5 ratings on a Likert scale from 1—not at all—to 5—a very great deal. The health-related QoL questions have a rating from 1 (none of the time) to 5 (all of the time). The 6 domains that constitute the QoL questions include concern, activities, energy/mood, control, self-consciousness, and sexual function [15,16,20,22]. There is an additional domain for overall QoL, referred to as health-related quality of life (HRQL).

The various clinical symptoms addressed by separate questions in the questionnaire included bleeding, pelvic pain, bulk symptoms, urinary frequency both during the daytime and nighttime, and fatigue. Note that the questionnaire was integrated into the patients’ electronic medical records (EMRs) in order to make it as easy as possible for the patients to complete them.

In every category and domain, the scores were summed, averaged, standardized, and transformed to a 100-point scale in order to obtain a final measure, expressed as a score out of 100. Because of the arithmetic involved in deriving such numbers, a lower number indicates improvement when dealing with clinical symptoms, whereas the opposite is true (i.e., a higher number) when assessing QoL.

### 2.3. Clinical and Imaging Evaluation and Uterine Artery Embolization Protocol

Variables such as the age of the patients, volume of the fibroids at baseline, the location and type of fibroids, and the size and number of vials of embolization particles were investigated and used as covariates for the data analysis. Note that every patient underwent either ultrasound or magnetic resonance (MR) imaging before UAE. These imaging studies were used to calculate the volume of the fibroids, and determine their location and type. In addition, clinical symptoms, including menorrhagia, menometrorrhagia, and the presence of blood clots and pelvic pain, were assessed before and after treatment in a binary fashion (i.e., whether such symptoms were present: yes or no). As part of the UAE procedure, which was standardized across all the sites, the fluoroscopy time in minutes and the access site into the artery (common femoral or radial artery) were also recorded, as they are included in the EMR. The particles used for embolization were tris-acryl gelatin microspheres measuring between 300 and 700 microns [11,12,13].

### 2.4. Statistical Analysis

A post-hoc analysis was conducted based on the UFS-QoL questionnaire and the treatment records. The comparison of means between two groups was performed using a two-tailed *t*-test. To compare the change in various QoL measures before and after the UAE treatment, we applied a two-tailed, paired *t*-test, whereas for comparisons between two groups with independent samples, e.g., women under vs. over 55 years of age, we ran a two-sample *t*-test. For correlation analysis of continuous variables, we used Pearson’s correlation. We avoided the Kendall and Spearman correlations because the ties in age and vial numbers could introduce ambiguity to the results. For example, we tested whether the patients’ age and the volume of their fibroids were correlated overall or conditional on the location of the fibroid. Among all the correlations we calculated, we only found the correlation between the volume of the fibroids and the total number of vials used to perform the UAE to be statistically significant. We used an ANOVA test for mean comparisons between groups, e.g., whether the volume of the fibroids varied across different locations, which was not significant (*p*-value = 0.11). For breaking down ages, we divided the patients into 3 age groups: under 45, between 45 and 55, and over 55 years of age. If the ANOVA test identified a significant difference regarding a QoL measure or a symptom between these age groups, the significance was then reported via a two-sided *t*-test for differences between these age groups, such as under 45 vs. over 55, and 45 to 55 vs. over 55. A one-sided Fisher’s exact test was used to assess binary changes in clinical symptoms (e.g., the presence or absence of menorrhagia before and after the UAE) before and after treatment. Alternatively, we used the chi-squared test, which incidentally matched the Fisher’s exact test outcome.

Finally, our analysis was performed using R version 4.3.2 and the “stats” package version 4.3.2 to calculate *p*-values and calculate other statistical measure. For graphs, we used the “ggplot2” package. We calculated Cohen’s d and its magnitude using the “rstatix” package as described in [23].

## 3. Results

**Figure 1:**
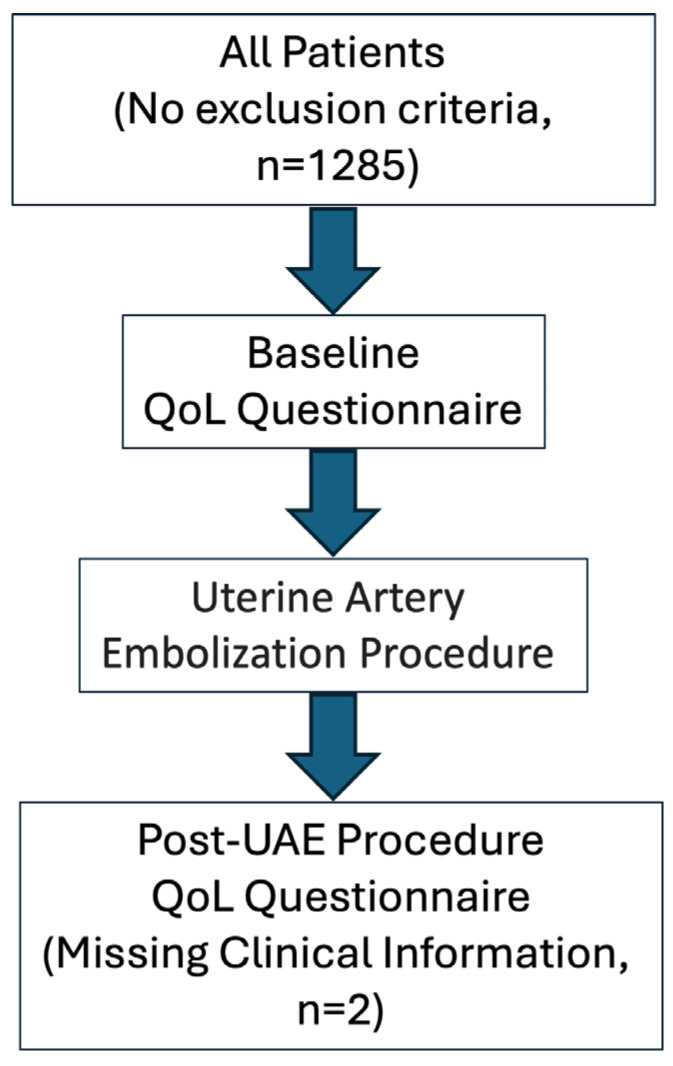
Flowchart of sample collection. No exclusions were applied.

A cohort of 1285 consecutive patients (average age: 44.5, standard deviation: 9.1) were treated with UAE and followed for an average time of 180 days post-treatment (patient characteristics shown in Table 1 and Figure 1). The fibroids were found to be intramural in 636 patients, subserosal in 416, and submucosal in 227 patients, whereas the type of fibroid was not recorded in 6 patients (Table 1). Every patient who came for the initial clinical visit and was treated with UAE returned between 2 and 8 months post-treatment for a clinical evaluation.

**Figure 2:**
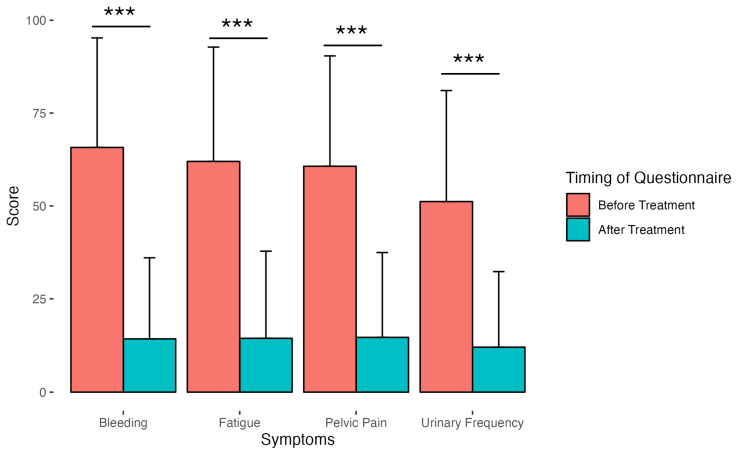
Clinical symptoms before and after UAE. The effect size of the four clinical subcategories pelvic pain, bleeding, urination frequency, and fatigue improved by 1.28, 1.45, 1.11, and 1.27, respectively. (Triple asterisks indicate *p*-value < 0.001.)

Most patients (95%) demonstrated a reduction in clinical symptoms, whereas a small number (5%) had worsening symptoms (Figure 2). More specifically, pelvic pain, bleeding, urinary frequency, and fatigue decreased in 94%, 96%, 92%, and 94% of patients, respectively (Figure 2). Among the 5% of patients who had worsening symptoms after the UAE, the mean score of their overall symptom severity increased by 16 points. Within that group, the mean score of each symptom measure also increased after the UAE, with urinary frequency being the worst at 24, followed by pelvic pain (13), fatigue (12) and bleeding (7) (all *p*-values < 0.001). Overall, within this small group of non-responders to UAE, 68% of the patients experienced worsening urinary frequency, whereas 50% reported worsening pelvic pain, fatigue, and bleeding.

**Figure 3:**
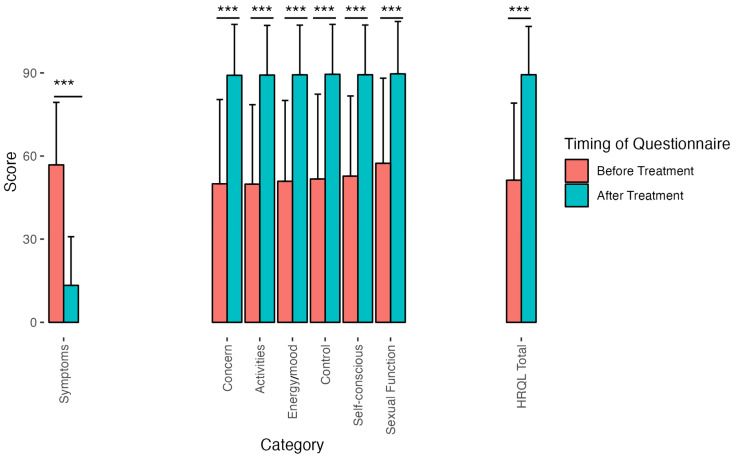
Overall clinical symptoms and QoL scores (out of 100) before and after treatment. All clinical symptoms and measures of QoL significantly improved after UAE. Note that, as mentioned previously, for symptoms, a decrease indicates an improvement, whereas for the QoL measures, the reverse is true. The absolute value of the effect size using Cohen’s d measure (mean change divided by pooled standard deviation) ranges from 0.96 (for sexual function) to 1.56 (for overall symptoms). (Triple asterisks indicate *p*-value < 0.001.)

**Figure 4:**
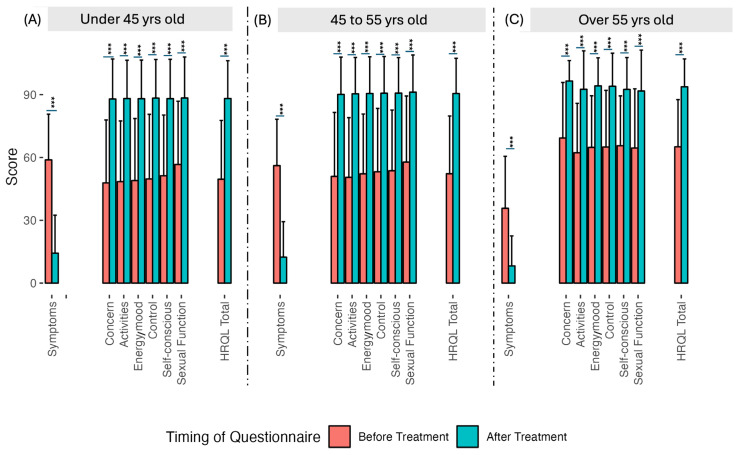
Clinical symptoms and QoL measures before and after UAE by age group: less than 45 years of age (**A**), between 45 and 55 years of age (**B**) and over 55 years of age (**C**). The over 55 age group had a better mean score than the under 45 and 45–55 age groups for symptoms and all QoL measures before treatment (*p*-value < 0.001 in all cases). After treatment, the mean score of the over 55 group remained better than the other two age groups; however, after UAE, the disparity between the age groups was reduced, and for most measures, the disparity lost its statistical significance. (Triple asterisks indicate *p*-value < 0.001.)

**Figure 5:**
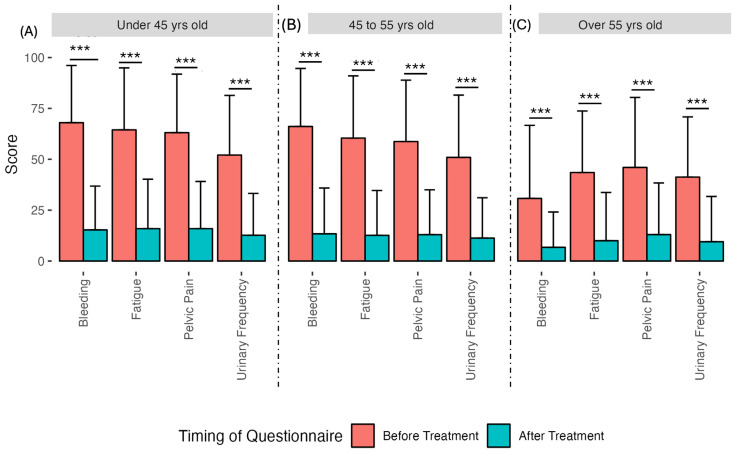
Various clinical symptoms before and after UAE by age group: less than 45 years of age (**A**), between 45 and 55 years of age (**B**) and over 55 years of age (**C**). As with Figure 4, at the baseline (before treatment), the over 55 age group had a better mean score in each clinical symptom category than the under 45 and 45–55 age groups. However, after treatment, the disparity between age groups decreased and lost its significance. (Triple asterisks indicate *p*-value < 0.001.)

Every QoL measure increased from baseline after UAE treatment, ranging from 90% for sexual function to 89% for activities (Figure 3). The overall health-related QoL (HRQL) increased by 89% after treatment (Figure 3). The overall symptom improvement for women under age 55 was statistically significantly better—with a mean of 44.17—than for women aged over 55, with a mean of 27.5 points (*p*-value < 0.0001, Figure 4A–C and Figure 5A–C). The overall HRQL for women under 55 also improved significantly more than for that of women over the age of 55 (mean of 38.44 vs. 28.68; *p*-value = 0.01, Figure 4A–C and Figure 5A–C). The number of vials used during UAE correlated well with the volume of fibroids pre-treatment (Pearson = 0.39, *p*-value ≤ 0.001), but it did not correlate with symptom improvement or any other QoL measure.

The location of the fibroids did not have any correlation with the QoL of the patients either before or after treatment. On the other hand, the type of fibroid (submucosal, subserosal, or intramural) did have a significant effect on patients’ clinical symptoms and most QoL measures (all ANOVA *p*-values < 0.05, except for the control). More specifically, before treatment, patients with submucosal fibroids had significantly worse QoL measures and experienced the most severe clinical symptoms (both *p*-values < 0.01). After UAE, differences between the submucosal and the other two fibroid types were not statistically significant both in terms of symptoms and QoL measures. Thus, post-UAE, the type of fibroids had no bearing on any of the clinical symptoms (*p*-value > 0.05 for all four categories).

The UAE procedures, which were performed in various OBL locations mostly in the northeast of the US, followed a standardized technique but did not mandate a specific arterial access between the common femoral and radial artery. The fluoroscopy time of the procedure, which was captured as part of the electronic medical records (EMRs) revealed that procedures performed through a femoral access took longer by almost 3 min than those performed through the radial artery (mean of 10 vs. 7 min, *p* < 0.001).

**Figure 6:**
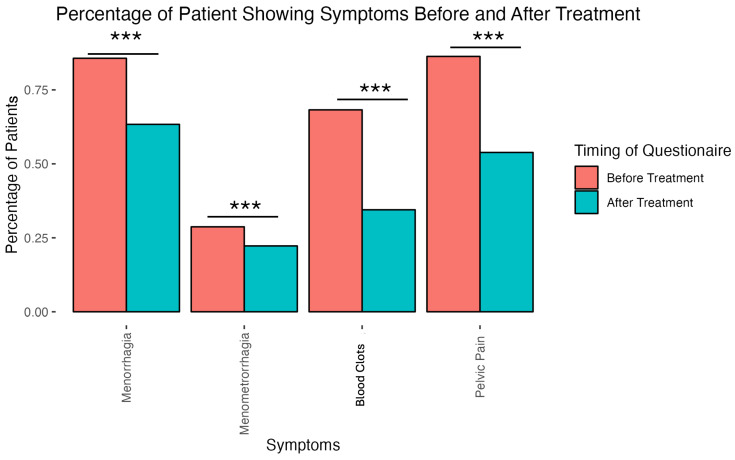
Percentage of patients reporting of clinical symptoms before and after UAE. Significantly fewer patients reported symptoms after the treatment. (Triple asterisks indicate *p*-value < 0.001.)

The presence or absence of clinical symptoms captured on the EMR in a binary fashion included menorrhagia, menometrorrhagia, blood clots, and pelvic pain. Resolution of these symptoms post-UAE was statistically significant (*p* < 0.001) (Figure 6).

## 4. Discussion

The results of our study—the largest study ever conducted on UAE—confirm the positive impact of UAE on the QoL of women affected by fibroid disease, even when the procedure is performed in various outpatient centers by different physicians. This was true across all measures included in the UFS-QoL questionnaire, including the eight questions designed to determine symptomatic responsiveness to the treatment and the twenty-nine health-related quality of life questions with six subscales designed to assess more generic health-related QoL issues. Women suffering from fibroid disease may hesitate to seek care to relieve their symptoms [7], and when they do, they are often told that hysterectomy is the preferred treatment, as the surgical removal of the uterus provides a definitive solution to their symptoms and essentially cures them of their ailment. The major drawback to hysterectomy for a benign indication lies in its aftermath, specifically how it negatively impacts the QoL of many women in both the short- and long-term, and how it may increase the risk of cardiovascular events, certain cancers, and early ovarian failure, as well as menopause [24]. Although UAE is not capable of the same “surgical” outcome as that of a hysterectomy, it is certainly capable of providing symptomatic relief in most cases, as has been shown in many studies [11,12,13,14,16,17,18]. Our results confirm such findings. Our patients, who were all seen in outpatient facilities in various areas of the United States by different physicians (interventional radiologists and vascular surgeons), were treated using the same exact clinical and procedural protocol. This rigor and standardization of care allowed us to achieve excellent and consistent outcomes. This was especially gratifying given the large number of patients treated in our study. Typically, our patients returned for a clinic visit at 3 and 6 months post-UAE to be clinically evaluated. The UFS-QoL questionnaire, which had been integrated into our EMR, was filled out both at baseline and during the follow-up clinic visits. While conducting our study, every one of our 1285 patients completed the two questionnaires at the time of the clinic visit, which is quite remarkable, allowing us to obtain the necessary data for analysis.

The main advantage of the UFS-QoL is that it addresses QoL issues that are specific to women affected by fibroid disease [15,16,20,21,22]. Furthermore, it has been validated across various treatments for fibroid disease, including UAE, myomectomy, hysterectomy, and others, such as radiofrequency thermal ablation, magnetic-resonance-guided ultrasound surgery, and treatment with medication [15,16,20,21,22]. Finally, the UFS-QoL is uniquely designed to not only assess physical or clinical symptomatology but also how such physical symptoms affect health-related QoL. This questionnaire is therefore ideally suited to evaluate the success of a particular therapy for fibroids. In our case, we were interested in determining whether the OBL setting where UAE was performed could negatively impact the clinical and QoL outcomes. It is reassuring that our patients did extremely well post-UAE based on every clinical and health-related QoL measure contained within the questionnaire. The observed reduction in bleeding, pelvic pain, bulk symptoms, urinary frequency, and fatigue is in keeping with those reported in the literature [16,21]. Similar observations were made about measures of health-related QoL, such as anxiety, resumption of life activities, mood, and sexual function. Our data unequivocally demonstrate the tremendous benefits of UAE regardless of the setting where the procedure was performed and, at a minimum, supports the concept of performing UAE in the outpatient setting, thereby cutting costs and providing improved access to patients—particularly those in low socioeconomic groups, who may be more hesitant to seek care for their fibroids. An interesting observation was that the number of vials used to embolize the uterine arteries during the procedures did not impact any of the clinical or health-related QoL outcomes, meaning that the angiographic endpoint—near stasis with five heartbeats to clear the contrast column—is far more meaningful than the number of vials. Such a finding confirms the importance of following a set and standardized procedural protocol to avoid inconsistencies in clinical outcomes.

Finally, it is important to note that more than one third of our patients had Medicaid insurance (39%) reflecting the relatively high proportion of patients coming from lower socioeconomic backgrounds. This is not surprising given the location of our centers, but it highlights the impact of our services on patient care by providing care to patients who might otherwise not have access to such high quality physicians.

The main limitation in our study results is the study’s reliance on a questionnaire, which always carries some degree of subjectivity, to ascertain the impact of UAE. However, unlike most other clinical studies using QoL questionnaires, ours was already validated and, most importantly, our study was conducted prospectively, which means every patient answered the questions during their clinic visits and did not have to recollect how they felt before the therapeutic intervention, thereby minimizing the recall bias that has plagued so many other QoL studies. Indeed, the absence of any recall bias provides additional validity and statistical meaning and strength to our study results.

Another potential limitation is the fact that we focused on a short-term assessment of QoL, with a mean of 180 days post-UAE. The first part of the COMPARE-UF registry, which also focused exclusively on short-term QoL outcomes following myomectomy, hysterectomy, and UAE [21], found no significant differences in QoL between patients in the myomectomy and UAE groups, whereas it found that hysterectomy provided the most profound relief. The key difference between our study and that of the COMPARE-UF is that our study was performed entirely in OBLs and we included patients with a broader range of ages, which allowed us to create the largest cohort to date of patients with fibroids treated with UAE. Because the COMPARE-UF study also used the UFS-QoL to assess QoL, meaningful comparisons between these two studies were possible. Using Cohen’s d, which made assessing differences between means and analyzing the effect size of each treatment possible, we found that our patients, who underwent UAE in the outpatient setting, had similar improvements in QoL to those who were treated with inpatient UAE or myomectomy in the COMPARE-UF registry (|d| < 0.2). Symptomatic relief was slightly better in our patient cohort than in the group treated with myomectomy (|d| = 0.37) but slightly worse than in the group treated with hysterectomy (|d| = 0.36) in the COMPARE-UF registry.

Since UAE is a uterus-sparing procedure, unlike hysterectomy, long-term outcomes post-UAE were found to be less durable than those post-hysterectomy in the latest report of the same COMPARE-UF registry [22]. This is the reason why our study was designed: to specifically address short-term QoL changes after UAE.

In summary, our prospective study confirms the benefit provided by UAE on the QoL of women affected by fibroid disease, and demonstrates for the first time that the outpatient setting does not negatively impact the QoL outcomes of UAE. Rather, in this largest-ever clinical study about the impact of UAE on the QoL of women with fibroid disease, every QoL measure was significantly and positively impacted by UAE. Since outpatient facilities provide easy accessibility to care, our results should therefore contribute to increasing treatment access for women of any socioeconomic group who have fibroids.

## Figures and Tables

**Table 1:** Patient characteristics. Note that, in some cases, covariates were not captured in the EMR, which resulted in undercounting in several subcategories.

*Age*				
	All Ages	<45	45–55	>55
Sample No	1284	721	513	50
Mean Age	44.48	39.81	49.22	58.54
*Laterality of Fibroids*				
Bilateral	207	114	87	6
Unilateral	1004	564	399	40
*Type of Fibroids*				
Intramural	596	331	242	23
Submucosal	217	138	77	2
Subserosal	392	205	166	20
*Location of Fibroids*				
Body	818	471	318	28
Cervix	24	14	7	3
Fundus	368	192	161	15
*Patient Group*				
Mean Volume	184.84	181.27	189.52	186.12
Sd Volume	84.73	88.37	79.80	81.47
*Arterial Access Site (Approach)*				
Femoral	628	366	244	17
Radial	348	187	148	13

## Data Availability

All data supporting the reported results are stored at USA Clinics due to privacy or ethical considerations.
